# Asthma diagnosis and treatment – 1005. Optimization for the withdrawal of inhaled corticosteroid treatment by monitoring fractional exhaled nitric oxide (feno) and lung functions

**DOI:** 10.1186/1939-4551-6-S1-P5

**Published:** 2013-04-23

**Authors:** Morimitsu Tomikawa, Kiyotake Ogura, Katsuhito Iikura, Noriyuki Yanagida, Sakura Sato, Takatsugu Komata, Akinori Shukuya, Yumi Koike, Motohiro Ebisawa

**Affiliations:** 1 Sagamihara National Hospital, Japan; 2 National Sagamihara Hospital, Japan; 3 Department of Pediatrics, National Sagamihara Hospital, Kanagawa, Japan; 4 Clinical Research Center for Allergy and Rheumatology, National Sagamihara Hospital, Kanagawa, Japan; 5 Department of Pediatrics, National Sagamihara Hospital, Sagamihara, Japan; 6 Department of Allergy, Sagamihara National Hospital, Clinical Research Center, Japan

## Background

There are no standardized criteria or protocol for the withdrawal of inhaled corticosteroids (ICS) from remitted pediatric asthma in Japanese Pediatric Guideline for the Treatment and Management of Asthma (JPGL).

## Methods

Among 55 asthmatic subjects in our hospital, FeNO and pulmonary functions were measured at the withdrawal of ICS, at 1 month and 3 month after. Those data were investigated whether it would be a predictor for the recurrence of asthma symptoms.

## Results

Subjects in recurrent asthma symptom group were 28 cases and those of non-recurrent asthma symptom group 27 cases (relapse rate: 50.9%). Any significant factors in background patients’ profiles, such as FeNO and pulmonary functions, were not associated with the recurrence of asthma. In recurrent asthma symptom group, FeNO was significantly increased by 3 months after withdrawal of ICS (from 31.8 ppb to 49.2 ppb). Among recurrent asthma symptom group, pulmonary functions were significantly decreased within 1 months (FVC: from 2.11L to 2.02L, FEV1.0: from 1.93L to 1.85L and %FEV1.0: from 98.1% of to 93.8%).

## Conclusions

Although these factors at the time of ICS withdrawal could not predict asthmatic revival, it is highly recommended to follow asthmatic patients who quit ICS therapy by measuring pulmonary function and FeNO periodically.

